# The prevalence of probable depression and probable anxiety, and associations with adverse childhood experiences and socio-demographics: A national survey in South Africa

**DOI:** 10.3389/fpubh.2022.986531

**Published:** 2022-10-28

**Authors:** Ashleigh Craig, Tamsen Rochat, Sara N. Naicker, Witness Mapanga, Asanda Mtintsilana, Siphiwe N. Dlamini, Lisa J. Ware, Justin Du Toit, Catherine E. Draper, Linda Richter, Shane A. Norris

**Affiliations:** ^1^South African Medical Research Council/Wits Developmental Pathways for Health Research Unit, Faculty of Health Sciences, University of the Witwatersrand, Johannesburg, South Africa; ^2^Department of Science and Innovation-National Research Foundation Centre of Excellence in Human Development, University of the Witwatersrand, Johannesburg, South Africa; ^3^Noncommunicable Disease Research Division, Wits Health Consortium (PTY) Ltd., Johannesburg, South Africa; ^4^School of Human Development and Health, University of Southampton, Southampton, United Kingdom

**Keywords:** adverse childhood experience (ACE), mental health, South Africa, national representative survey, probable depression, probable anxiety

## Abstract

**Objective and methods:**

Mental health problems among adults are a growing public health concern, and middle-income countries such as South Africa are disproportionally affected. Using a large scale nationally representative weighted survey, we assessed the prevalence of probable depression, probable anxiety, and adverse childhood experiences (ACEs), and explored associations between probable depression, probable anxiety, ACEs, socio-economic status, and demographic characteristics.

**Results:**

Nationally, 25.7, 17.8, and 23.6% of respondents, respectively, reported scores of ≥10 on the Patient Health Questionnaire-9 (PHQ-9) and Generalized Anxiety Disorder-7 (GAD-7), indicating probable depression or probable anxiety, and an ACE score of ≥4 (high exposure). Overall probable depression prevalence across South Africa varied from 14.7 to 38.8%. Both probable depression and probable anxiety were more frequently reported among adults who were: retired and older (>65 years of age), and widowed, divorced, or separated; living in metropolitan areas; and only had primary school education. In a multivariable adjusted logistic regression, the likelihood of reporting probable depression or probable anxiety was also found to increase with each standard deviation increase in the ACE score (*p* < 0.001), independent of other socio-demographic determinants.

**Conclusion:**

The prevalence of probable depression among respondents in South Africa varies significantly across the nine provinces. Furthermore, higher ACE score and several socio-demographic determinants were associated with a higher likelihood of probable depression and probable anxiety. Adult mental health services are urgently needed to identify groups of the population vulnerable to mental health problems for better targeting of interventions. Given the range of probable depression prevalence across the country, provincial level plans and resources should also reflect the burden of mental health problems in that province.

## Introduction

Mental health problems among adults in low- or middle-income countries is an ever-increasing public health concern (1). In a 2009 study in South Africa, a middle-income country with significant income disparity (2), nearly 20% of adults were reported to suffer from impaired mental health, with less than a quarter of this population ever seeking mental health treatment (3). In 2013, the highest lifetime prevalence rate of depression was reported in a study in the Eastern Cape (31.4%), one of the poorest provinces in South Africa (4). Another South African study conducted in 2018 in urban informal settlements found that nearly one in every five women reported moderate to severe levels of anxiety (5). It has also been reported that more than half of South African adults have been exposed to adverse childhood experiences (ACEs) such as emotional or sexual abuse during childhood, with about 40% having experienced some sort of emotional neglect before the age of 18 years (6).

Certainly, several factors are known to influence the development of depression, such factors include anxiety (7), early adversity (8, 9), socio-economic status and demographic characteristics (10–13). Due to fear, uncertainty and social and economic disruptions associated with the COVID-19 pandemic, the prevalence of mental illness in South Africa, as in other countries (14), may have worsened. Findings from a recent study in a smaller urban setting demonstrated that adults with a history of early adversity were more likely to experience depressive symptoms, particularly when at a high risk for COVID-19 infection (15). Evidence for the substantial impact of COVID-19 on mental health in South Africa was also documented as early as October 2020, just a few months after the initial lockdown (March 2020), with the authors suggesting likely implications on future mental health for South Africans (16). Considering the inequalities in South Africa, taken with the economic and social turmoil of the pandemic, individuals living in poverty and with poor mental health are at increased risk of remaining impoverished due to difficulties with learning and earning, higher health expenses and discrimination (17).

Mental illness significantly impairs overall health (18) with the comorbidity of depression and anxiety being reported in numerous settings (19, 20). Likewise, higher ACE scores can—independent of other risk factors—identify groups of individuals at heightened mean risk of poor health in later life (21). This large-scale, nationally representative study, therefore, assessed the prevalence of mental health problems among South African adults (post COVID-19 wave 3) and explored socio-economic and demographic associations with probable depression, probable anxiety, and ACEs. This study provides important evidence for understanding determinants that are strongly linked to probable depression and probable anxiety outcomes, which may assist in identifying mechanisms of these associations, and aid in developing targeted intervention strategies to reduce the risk of poor mental health in South Africa. The data was also disaggregated by province to inform provincial service provision and prevention.

## Materials and methods

**Figure 1:**
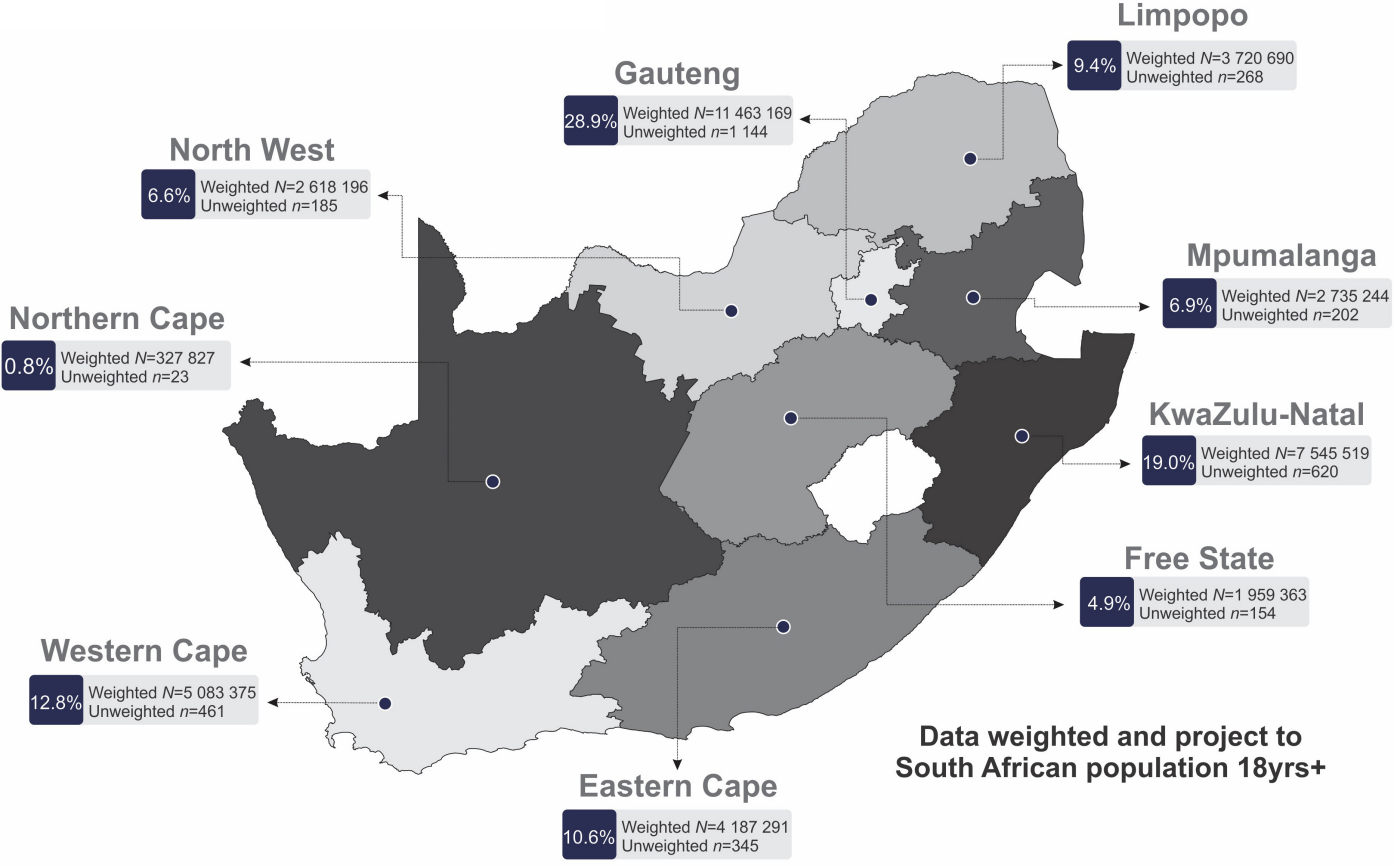
Population demographics outlining the nine provinces of South Africa.

**Figure 2:**
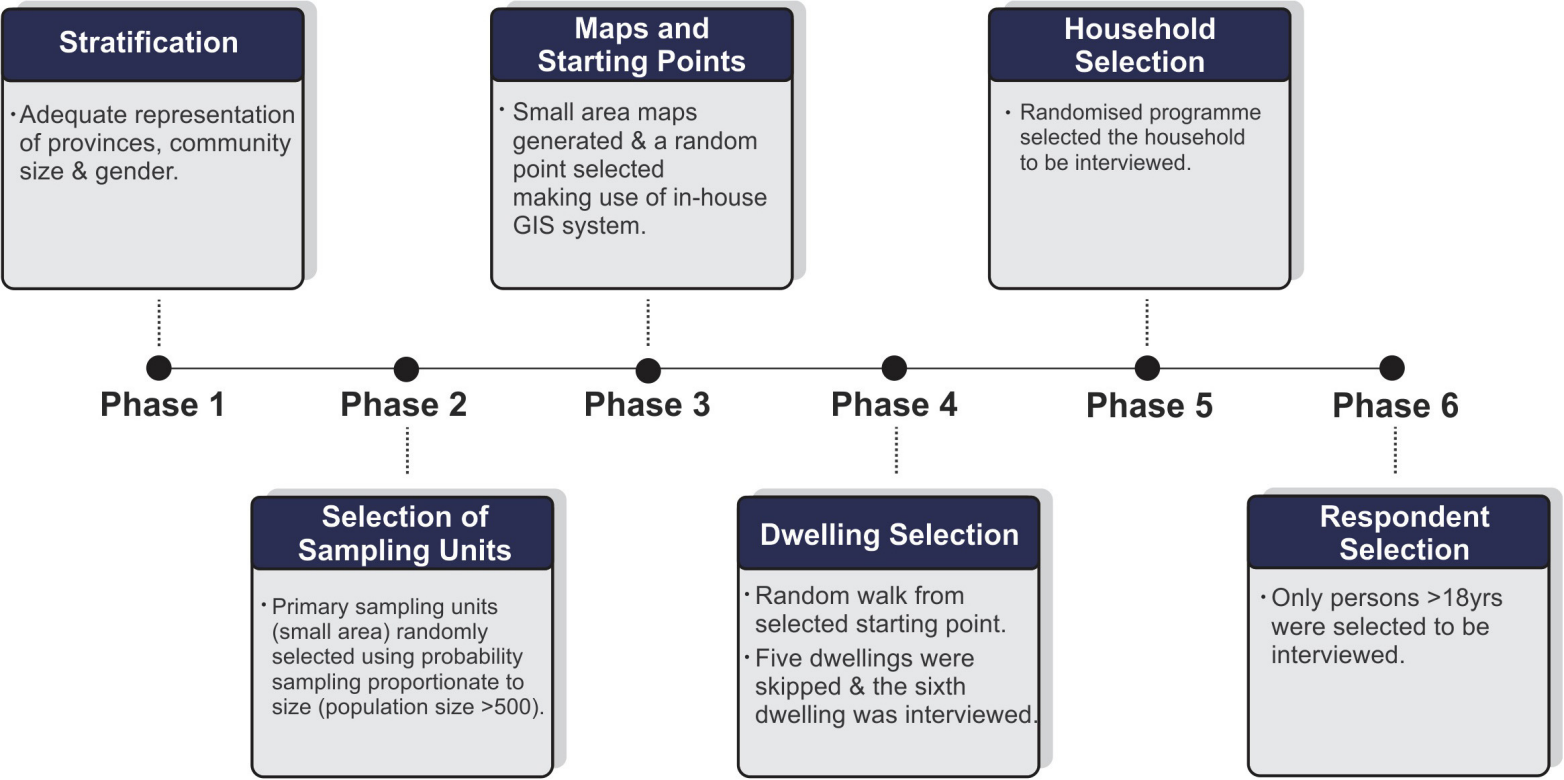
Schematic illustration of the six phases of data collection. GIS, Geographical Information System.

This cross-sectional study surveyed a nationally representative sample of adults (>18 years old). Data collection commenced on the 3rd September and concluded on the 29th October, 2021 (10 weeks). Data collection was carried out by a team of 180 experienced fieldworkers across the nine provinces of South African drawing upon established research infrastructure and staff coordination system. All fieldworkers went through extensive training on the survey items (item understanding, language proficiency, explanations to participants, questionnaire flow) with mock interviews prior to commencement of data collection to ensure uniform understanding and execution of the survey. Retraining occurred for field staff that required additional support identified through daily back checking and fieldwork coordinators. Interviews were conducted with 3,402 participants across the nine provinces of South Africa (Figure 1). Data collection was obtained in a six-phase approach with the use of a stratified probability sampling method as outlined below (Figure 2).

In phase 1, we identified community sizes (metropolitan areas, cities, large and small towns, large villages, and rural areas) and gender distribution to ensure adequate representation across provinces. Phase 2 consisted of randomly selected sampling units (defined as small areas) where six interviews (minimum recommend number statistically) per small area would be conducted. Phase 3 made use of mapping technology (GIS) to identify a starting location. Once a starting point was identified, phase 4 proceeded with the first household being interviewed. Thereafter, five houses were omitted, and the sixth household was interviewed. In South Africa, the term household refers to either one person living alone or a group of people—usually, but not always, members of one family who live together for at least four nights a week, and whose food and other household expenses are managed as one unit. In phase 5 interviewers requested a list of all households residing in the dwelling, after which a built-in randomized program selected specific households to be interviewed. This was based on the total number of households in the dwelling. Finally, in phase 6, an automated Kish grid selected the respondent in the identified household who were older than the age of 18 years to be interviewed for data collection.

The study obtained approval from the Human Research Ethics Committee (Non-Medical) of the University of the Witwatersrand, South Africa (H21/06/36). Written informed consent was obtained from each respondent.

### Survey

Data collection took place through face-to-face interviews to administer a survey with the use of computer assisted personal interviewing technology. The survey included questions related to respondent and household demographics. Province and community size (metropolitan, city/town, rural/village) were recorded and information collection on household assets, age, gender, employment status, marital status, education attained, and mental health. A household asset score—an indicator of socio-economic status—was computed in alignment with the Demographic and Health Surveys household questionnaire. This included a tally of all major operational household amenities (e.g., refrigerator, washing machine, television, computer etc.). In this cross-sectional study, household asset score tertiles were computed and used as an indicator of economic differentiation (22–24).

Due to the nature of the survey, mental health risk was based on the presence of symptoms and not on diagnoses made by a trained mental health professional. As such, depression and anxiety as an outcome will be referred to as probable depression and probable anxiety throughout this study.

To assess probable depression, the Patient Health Questionnaire (PHQ-9) scale was used (25). This scale consists of 9 questions with responses recorded on a four-point Likert scale ranging from 0 (“Not at all”) to 3 (“Nearly every day”). The level of probable depression was then categorized into five groups, which were, minimal, mild, moderate, moderately severe, and severe based on scoring in the range of 0–4, 5–9, 10–14, 15–19, and 20–27, respectively (25). The PHQ-9 survey has been clinically validated with a sensitivity of 88.0% and a specificity of 88.0% at a cut-off score of 10 or higher (25). Therefore, binary classification of probable depression was defined by a PHQ-9 score of 10 or greater.

Similarly, to assess probable anxiety, the Generalized Anxiety Disorder (GAD-7) scale was used (25). The GAD-7 scale consists of 7 questions with a four-point Likert response scale ranging from 0 (“Not at all”) to 3 (“Nearly every day”). The level of probable anxiety was categorized into four groups, which were minimal, mild, moderate, and severe based on scoring in the range of 0–4, 5–9, 10–14, and 15–21, respectively (25). Binary classification of probable anxiety was defined by a GAD-7 score of 10 or greater (26).

Adverse childhood experiences (ACEs) were measured through a 12-item ACE questionnaire (Supplementary Table 1), which is an individual's retrospective report of specific adversities experienced over the first 18 years of their life (27). Exposure to ACEs was operationalized using 12 types of experiences falling within three categories: emotional and/or physical abuse, sexual abuse, or household dysfunction (27). An overall ACE score was calculated based on the number of affirmative responses (yes response) to the binary 12 ACE questions (no = 0/yes = 1). The accumulative ACE score was categorized into 3 exposure groups based on scoring 0 (low exposure); 1–3 (intermediate exposure) and 4–12 (high exposure), respectively (28).

### Statistical analyses

**Figure 3:**
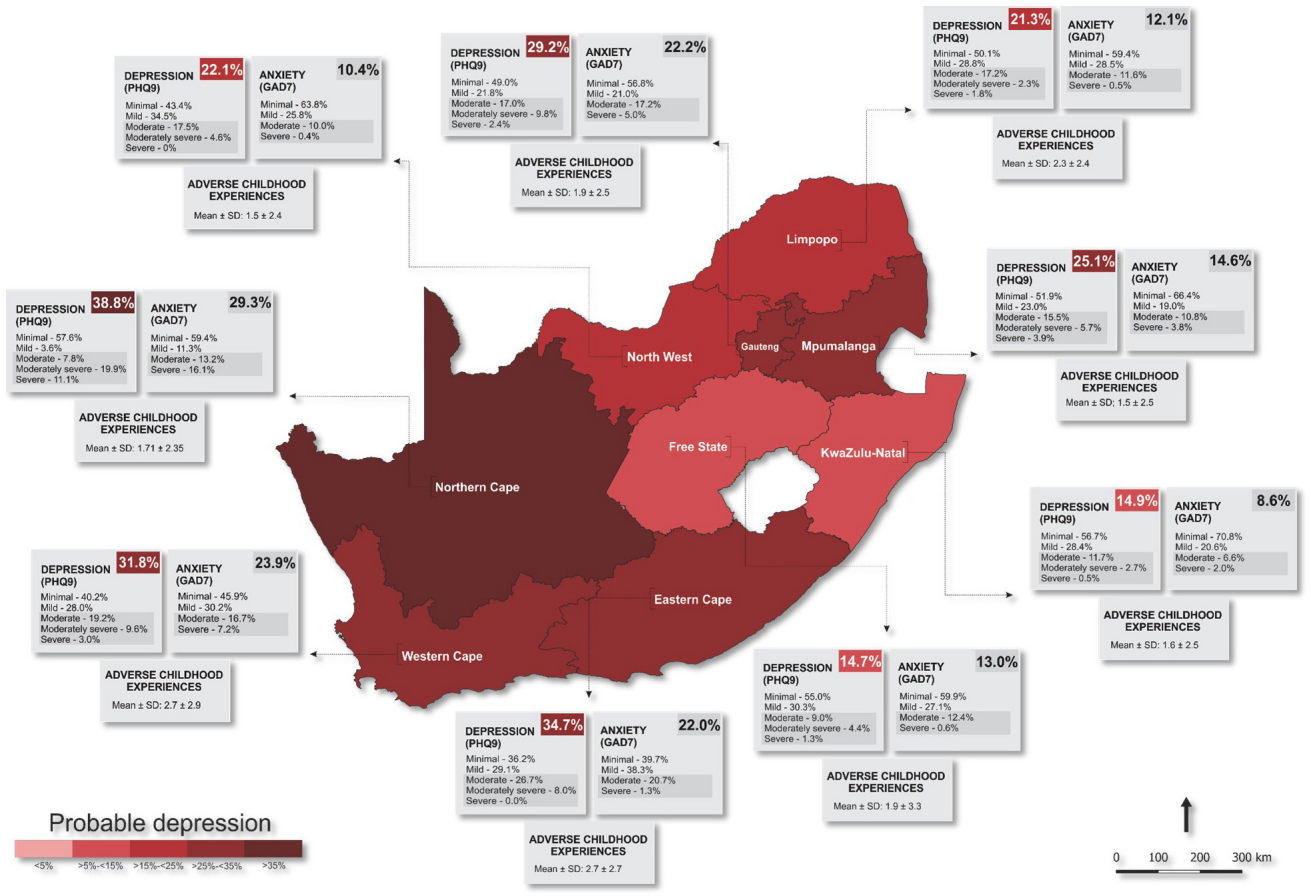
Prevalence of mental health risk across South Africa. PHQ9, Patient Health Questionnaire; GAD7, Generalized Anxiety Disorder.

For all statistical analyses, IBM^®^ SPSS^®^ version 28 (IBM Corporation, Armonk, New York) and GraphPad Prism version 5.03 for Microsoft^®^ Windows (GraphPad Software, San Diego, California, USA) were used to analyze and plot the data. Additionally, QGIS (Penn Libraries, Philadelphia, PA) was used to plot and scale the geographical location of the South African provinces (Figures 1, 3).

All statistics were weighted to represent the most recent census of the South African population (>15 years of age). The weighted matrix factored in age, sex, population group, home language and provincial distribution. Proportions across socio-demographics (age, marital status, education level, employment, household assets and urbanicity) and provinces were determined with crosstabs with significant differences indicated by Chi-square tests and presented as percentages. Univariate and multivariable adjusted binary logistic regressions were performed to determine the odds of probable depression (PHQ-9 score ≥ 10) and probable anxiety (GAD-7 score ≥ 10) in adulthood with either ACEs or ACEs and socio-demographic contributors (age, sex, race, marital status, education level, employment, household assets and urbanicity) as confounders. Additionally, linear regression analyses were conducted with probable depression score, or probable anxiety score as continuous dependent variables and tested separately for their association with ACEs or, ACEs and a combination of socio-demographic contributors. In all statistical analyses, probable depression, and probable anxiety (continuous or binary) were considered dependent outcome variables, while ACE score and/or socio-demographic determinants were considered independent variables in the various models.

## Results

In total, 3,402 respondents (female: 50.8%; male: 49.2%) were included in the final analyses (Table 1). Respondents were predominantly adults under the age of 44 years (18–24 years: 13.2%; 25–34 years: 25.4%; 35–44 years: 30.8%; 45–54 years: 19.0%; 55–64 years: 8.5% and 65 years or older: 3.0%). The largest proportion of respondents were those who reported a marital status of single (57.1%), employed (60.7%), an education level of graduated high school or equivalent (49.2%), and reside in the Gauteng province [*n* = 1,144 (28.9%)] (Figure 1).

**Table 1:** General descriptives of the South African survey respondents (*n* = 3,402).

		**Unweighted**	**Weighted**			**Unweighted**	**Weighted**
**Age categories**				**Probable depression categories**	
18–24 years	%	15.0	13.2	Minimal	%	49.1	48.2
25–34 years	%	27.4	25.4	Mild	%	26.2	26.1
35–44 years	%	29.6	30.8	Moderate	%	16.4	16.9
45–54 years	%	17.3	19.0	Moderately severe	%	6.8	7.0
55–64 years	%	8.0	8.5	Severe	%	1.5	1.8
65+ years	%	2.6	3.0	Probable depression	%	24.7	25.7
**Education**				**Probable anxiety categories**	
Uneducated/partial primary	%	1.8	1.8	Minimal	%	58.1	57.3
Primary school	%	1.5	1.5	Mild	%	25.2	24.9
Partial secondary	%	18.0	18.7	Moderate	%	13.4	14.2
NSC/short course	%	50.2	49.2	Severe	%	3.3	3.6
Tertiary	%	28.5	28.8	Probable anxiety	%	16.7	17.8
**Sex**				**ACE score**	
Male	%	49.2	49.4	Low exposure (0)	%	47.8	47.0
Female	%	50.8	50.6	Intermediate exposure (1–3)	%	28.9	29.4
				High exposure (4+)	%	23.3	23.6
**Employment status**				**Household assets**	
Unemployed	%	27.1	27.5	Lower tertile	%	35.0	34.9
Employed	%	60.8	60.7	Middle tertile	%	35.8	35.4
Student	%	7.3	6.4	Upper tertile	%	29.2	29.7
Retired	%	4.8	5.3				
**Marital status**				**Urbanicity**	
Single	%	57.6	57.1	Metropolitan	%	53.6	54.5
Married/co-habit	%	32.8	33.3	City/town	%	23.8	23.2
Widowed/divorced	%	9.6	9.6	Rural/village	%	22.5	22.3

### Prevalence of mental health problems

Overall, the majority of respondents had a minimal risk PHQ-9 score (48.2%), while those scoring as mild, moderate, moderately severe, and severe were 26.1, 16.9, 7.0, and 1.8%, respectively, which resulted in a prevalence of 25.7% of probable depression in South Africa (moderate, moderately severe, and severe) (Table 1). Northern Cape reported the highest prevalence of both probable depression (38.8%) and probable anxiety (29.3%) out of all nine provinces (Figure 3; Supplementary Table 2). Free State province reported the lowest prevalence of probable depression (14.7%), while KwaZulu-Natal reported the lowest prevalence of probable anxiety (8.6%). Furthermore, ACE scores were recorded in the range of 1.5–2.7, with the highest mean ACE score (2.7), reported in both the Western and Eastern Cape provinces (SD ≤1.7).

### Socio-demographic correlates

**Figure 4:**
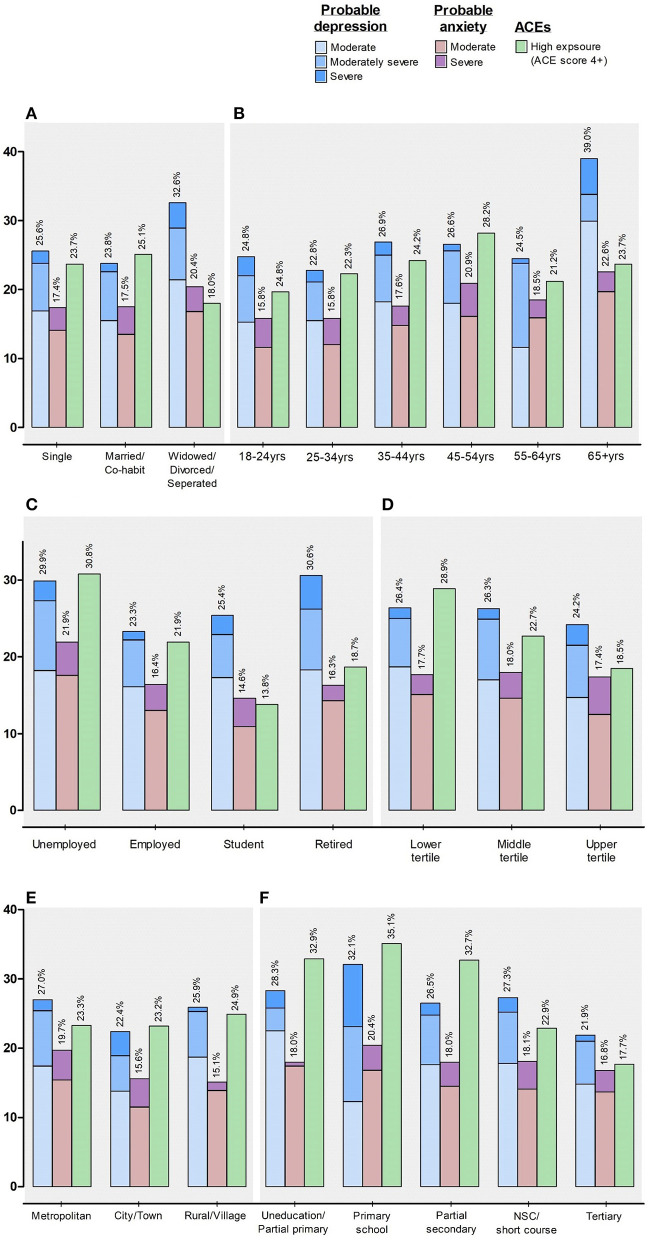
Mental health risk stratified by **(A)** marital status; **(B)** age categories, **(C)** employment status, **(D)** household asset score, **(E)** urbanicity, and **(F)** education level. %, percentage; NSC, National Senior Certificate (Grade 12).

Figure 4 (Supplementary Table 3) presents the prevalence of probable depression, probable anxiety, and ACEs among the study population stratified by socio-demographic characteristics. The prevalence of probable depression was highest among women (26.7%), widowed, divorced, or separated respondents (32.6%), and those who were retired (30.6%), 65 years or older (39.0%) and/or with a household asset score in the lowest tertile (26.4%). Additionally, probable depression was higher among respondents residing in the metropolitan area (27.0%) and/or with only a basic level of education, i.e., completed primary school (32.1%).

The highest prevalence of probable anxiety (Figure 4; Supplementary Table 3) was among respondents who reported a marital status as widowed, divorced or separated (20.4%), and employment status of unemployed (21.9%), 65 years or older (22.6%), with a household asset score in the middle tertile (18.0%). Similar to those respondents who reported the highest probable depression, respondents with the highest probable anxiety also resided in the metropolitan area (19.7%), and/or only completed primary school (20.4%).

Upon categorizing those respondents according to their accumulated ACE scores, those in the highest exposure groups with an ACE score of 4 or greater (Figure 4; Supplementary Table 3) were respondents who were married or co-habit (25.1%), in the age range of 45–54 years (28.2%), currently unemployed (30.8%), or with a household asset score in the lowest tertile (28.9%). Furthermore, nearly a quarter of these respondents reported living in rural settings (24.9%), again with only a basic level of education (35.1%).

### Associations of probable depression, probable anxiety, ACE score, and socio-demographics

We performed univariate and multivariable adjusted binary logistic regressions (Table 2A) to determine the odds of having either probable depression or probable anxiety with higher levels ACE exposure (model 1), or having ACE exposure, independent of socio-demographic characteristics (age, marital status, education level, employment, household assets and urbanicity) (model 2). In model 1, we determined that, the likelihood of having probable depression increased by 22% [OR, 1.22 (95% CI 1.217; 1.217)] with each standard deviation increase in the ACE score (*p* < 0.001). Similarly, the likelihood of having probable anxiety increased by 21% [OR, 1.21 (95% CI 1.208; 1.209)] with each standard deviation increase in the ACE score (*p* < 0.001). Furthermore, in model 2, the odds of either probable depression or probable anxiety increased with each standard deviation increase in the ACE score (*p* < 0.001), independent of other socio-demographic determinants.

**Table 2A:** Logistic regressions to determine the odds of having probable depression or probable anxiety in adulthood.

			**Probable depression (PHQ-9)** **Binary variable** **(*****n*** = **3,402)**			**Probable anxiety (GAD-7)** **Binary variable** **(*****n*** = **3,402)**		
			**OR**	**(95% Cl)**	***p-*value**	**OR**	**(95% Cl)**	***p*-value**
Model 1	ACE	Score	1.22	(1.217; 1.217)	**<0.001**	1.21	(1.208; 1.209)	**<0.001**
Model 2	ACE	Score	1.20	(1.194; 1.195)	**<0.001**	1.19	(1.193; 1.194)	**<0.001**
	Age	Years	1.00	(1.003; 1.003)	**<0.001**	1.01	(1.007; 1.008)	**<0.001**
	Sex	Male	(Reference)			(Reference)		
		Female	1.06	(1.060; 1.064)	**<0.001**	1.04	(1.034; 1.038)	**<0.001**
	Race	Black	(Reference)			(Reference)		
		White	1.20	(1.194; 1.202)	**<0.001**	0.920	(0.917; 0.924)	**<0.001**
		Indian/Asian	0.909	(0905; 0.914)	**<0.001**	0.693	(0.689; 0.697)	**<0.001**
		Colored	1.23	(1.226; 1.232)	**<0.001**	1.04	(1.041; 1.047)	**<0.001**
	Education	Uneducated/partial primary	(Reference)			(Reference)		
		Primary	1.29	(1.281; 1.303)	**<0.001**	1.25	(1.233; 1.258)	**<0.001**
		Partial secondary	1.15	(1.147; 1.161)	**<0.001**	1.20	(1.192; 1.210)	**<0.001**
		NSC/short course	1.44	(1.435; 1.453)	**<0.001**	1.39	(1.381; 1.402)	**<0.001**
		Tertiary	1.09	(1.083; 1.098)	**<0.001**	1.27	(1.262; 1.282)	**<0.001**
	Household assets	Score	1.01	(1.012; 1.012)	**<0.001**	1.03	(1.033; 1.034)	**<0.001**
	Employment	Unemployed	(Reference)			(Reference)		
		Employed	0.811	(0.809; 0.812)	**<0.001**	0.755	(0.754; 0.757)	**<0.001**
		Student	0.988	(0.985; 0.992)	**<0.001**	0.837	(0.834; 0.841)	**<0.001**
		Retired	1.04	(1.034; 1.042)	**<0.001**	0.643	(0.640; 0.646)	**<0.001**
	Marital status	Single	(Reference)			(Reference)		
		Married/co-habit	0.839	(0.838; 0.841)	**<0.001**	0.856	(0.854; 0.858)	**<0.001**
		Widowed/divorced/separated	1.43	(1.424; 1.432)	**<0.001**	1.23	(1.228; 1.236)	**<0.001**
	Urbanicity	Metropolitan	(Reference)			(Reference)		
		City/towns	0.715	(0.714; 0.717)	**<0.001**	0.741	(0.740; 0.743)	**<0.001**
		Rural/village	0.776	(0.775; 0.778)	**<0.001**	0.572	(0.570; 0.574)	**<0.001**

Model 1: regression unadjusted.

Model 2: regression adjusted for socio-demographics.

n, number of participants.

Bold values denote statistical significance (p < 0.05).

In linear regression analyses, we found expected positive associations (Table 2B) between probable depression (adj. *R*^2^ = 0.100; β = 0.674; *p* < 0.001) or probable anxiety (adj. *R*^2^ = 0.113; β = 0.598; *p* < 0.001) and ACE score (model 1). Additionally, both probable depression (adj. *R*^2^ = 0.099; β = 0.619; *p* < 0.001) and probable anxiety (adj. *R*^2^ = 0.114; β = 0.564; *p* < 0.001) scores associated positively with ACE score, after adjustments for several socio-demographic confounders.

**Table 2B:** Standard multiple linear regression with probable depression score and probable anxiety score as dependent variables.

			**Probable depression (PHQ9) Overall score** **(*****n*** = **3,402)**			**Probable anxiety (GAD7)** **Overall score** **(*****n*** = **3,402)**		
			**Adj *R*^2^**	**β (95% Cl)**	***p*-value**	**Adj *R*^2^**	**β (95% Cl)**	***p*-value**
Model 1	ACE	Score	0.100	0.674 (0.673; 0.674)	**<0.001**	0.113	0.598 (0.597; 0.598)	**<0.001**
Model 2	ACE	Score	0.099	0.619 (0.618; 0.620)	**<0.001**	0.114	0.564 (0.564; 0.565)	**<0.001**
	Age	Years		−0.001 (−0.001; −0.001)	**<0.001**		0.009 (0.009; 0.009)	**<0.001**
	Sex	Male	(Reference)			(Reference)		
		Female		0.338 (0.334; 0.341)	**<0.001**		0.158 (0.155; 0.161)	**<0.001**
	Race	Black	(Reference)			(Reference)		
		White		0.441 (0.434; 0.448)	**<0.001**		0.141 (0.135; 0.147)	**<0.001**
		Indian/Asian		−0.710 (−0.720; −0.700)	**<0.001**		−0.183 (−0.192; −0.175)	**<0.001**
		Colored		0.046 (0.040; 0.052)	**<0.001**		0.356 (0.351; 0.360)	**<0.001**
	Education	Uneducated/partial primary	(Reference)			(Reference)		
		Primary		0.849 (0.831; 0.868)	**<0.001**		−0.748 (−0.764; −0.732)	**<0.001**
		Partial secondary		−0.321 (−0.334; −0.307)	**<0.001**		−0.218 (−0.230; −0.207)	**<0.001**
		NSC/short course		0.002 (−0.011; 0.016)	0.76		−0.242 (−0.253; −0.230)	**<0.001**
		Tertiary		−0.282 (−0.296; −0.268)	**<0.001**		−0.132 (−0.143; −0.120)	**<0.001**
	Household assets	Score		−0.007 (−0.008; −0.007)	**<0.001**		0.022 (0.022; 0.023)	**<0.001**
	Employment	Unemployed	(Reference)			(Reference)		
		Employed		−0.696 (−0.700; −0.691)	**<0.001**		−0.511 (−0.514; −0.508)	**<0.001**
		Student		−0.276 (−0.284; −0.268)	**<0.001**		−0.452 (−0.459; −0.446)	**<0.001**
		Retired		0.131 (0.121; 0.140)	**<0.001**		−0.386 (−0.394; −0.379)	**<0.001**
	Marital status	Single	(Reference)			(Reference)		
		Married/co-habit		−0.558 (−0.562; −0.553)	**<0.001**		−0.610 (−0.614; −0.607)	**<0.001**
		Widowed/divorced/separated		0.448 (0.442; 0.455)	**<0.001**		0.091 (0.085; 0.096)	**<0.001**
	Urbanicity	Metropolitan	(Reference)			(Reference)		
		City/towns		−0.281 (−0.285; −0.276)	**<0.001**		−0.351 (−0.355; −0.348)	**<0.001**
		Rural/village		−0.250 (−0.255; −0.245)	**<0.001**		−0.657 (−0.661; −0.653)	**<0.001**

Model 1: regression unadjusted.

Model 2: regression adjusted for socio-demographics.

n, number of participants.

Bold values denote statistical significance (p < 0.05).

## Discussion

Data from this national study suggests a widespread prevalence of mental health problems across the South African adult population. More than a quarter of South African respondents reported moderate to severe symptoms of probable depression. In comparison with other national surveys conducted in low- and middle-income countries (LMICs), probable depression in South Africa is in line with a previous national survey [Peru: 23.2 % in 2020 (29)], however, is more than double that reported in another [Brazil: 7.9% in 2016 (30)]. Our survey also suggests that 17.8% of respondents reported probable anxiety (GAD-7 score ≥ 10) and 23.6% of respondents reported high-exposure ACE scores (≥4). Our data showed that probable depression, probable anxiety, and ACE prevalence varied across the nine provinces in the country, supporting earlier studies, which reported that differences in mental health risk exist across geographical areas of South Africa (13, 31).

Based on previous literature presenting evidence that mental health risk has significant associations with poverty (18, 32) and with higher prevalence previously reported in rural areas (33, 34), this could have resulted in the diverse prevalence of mental health risk we report across the provinces. I.e., the Eastern Cape province is reported to be one of the poorest provinces in South Africa (2019), with a total of 67.3% of adults living below the poverty line (35). Therefore, the ACE prevalence in the Eastern Cape was thus expected. We were, however, surprised to see the heightened ACE prevalence in the Western Cape province—one of the richest of the nine provinces in South Africa—and presumably the one province that also has had the least reconstruction to do post-apartheid (36–38). However, distance is one of the biggest barriers to all varieties of access to health care in the Western Cape (36–38). Therefore, we can only speculate that the ACE prevalence is highest in the Western Cape province due to a large proportion of the province lacking sufficient access to health care services (36–38). Likewise, it is known that depression is also strongly associated with poverty (32). Poverty and unemployment in South Africa are often rural phenomena and given that many of the rural inhabitants are linked to agricultural activities, the Northern Cape province may have reported the highest prevalence of probable depression due to this province accounting for the largest share of the South Africa's commercial agricultural land (37.1%) (39).

This study found a significant association between ACE scores with probable depression and probable anxiety outcomes across various models, even after adjusting for socio-demographic factors. Both probable depression and probable anxiety were associated with increased odds of ACE exposure. The fact that both probable depression and probable anxiety were associated with increased odds of ACE exposure is consistent with previous research pointing to an association between the degree of anxiety and depression, thus emphasizing the concomitance of anxiety and depression (40). From our data, 58.1% of respondents with probable depression also reported probable anxiety which further highlights the comorbidity of these two mental health conditions (40). Additionally, our finding of probable depression or probable anxiety likely increasing by 22 or 21% respectively, with each standard deviation increase in ACE score, is in line with previous literature stating that the early years of life are crucial in determining mental health outcomes in later life (41, 42). These findings therefore confirm previous studies that suggest reported ACEs are associated with an increased risk of mental health problems in adulthood (43, 44). This further highlights the importance of universal and population-based interventions in early childhood (41, 42), especially large-scale population-based surveys of the nature where the prevalence of exposure to ACEs is necessary; this could also determine how ACEs cluster within contexts which would aid in response. Comprehensive, integrated family- and community-center policies are required that not only stretch over government departments such as Health, Education and Social Development but, are also rooted in frameworks like the Nurturing Care Framework, which promotes a safe, secure, and nurturing environment in childhood (45). Social services should be trained to deliver their services as an adversity- and trauma-informed workforce.

Reports of probable depression, probable anxiety, and ACEs differed markedly across several socio-demographic determinants, such as marital status, age, education level, and living circumstances. Our evidence suggests that these factors increase the frequency and extent of depression independently of one another. Both probable depression and probable anxiety were significantly higher among respondents who reported being widowed, divorced, or separated than among those who reported being single, married or co-habiting (by >7%), which is similar to that reported by several other studies (46, 47). Likewise, numerous longitudinal studies have shown separation and divorce are associated with a high prevalence of depression (48–51), presumably due to financial and psychosocial stress related to marital disruption (52).

In recent years, studies of depression in later life have rapidly increased as its extent and etiology in older individuals are more fully explored (53). The prevalence of clinically significant depression at any given time in community samples of adults aged 65 years and older ranges from 9.8 to 38.0%, respectively (53–55), which is comparable to the finding in our 65-year and older age category (39.0%). Depression in later life is frequently seen as comorbid with several physiological and psychological conditions such as myocardial infarction (56), diabetes (57), stroke (58), and Alzheimer's disease (59). The aging process itself results in diverse changes in overall health which, over time, may alter an individual's self-image and esteem, autonomy, and overall functionality (60). Likewise, the prevalence of depression is more frequent among retirees (61), as changes in lifestyle adopted during many years in the workplace may alter an individual's psychosocial behavior (62). Additionally, many individuals understand the retirement process as one by which they start to become inactive (63), potentially generating feelings of uselessness and predisposing older adults to higher mental health risk.

Uneducated adults or those with little education vs. those who are better educated are at increased risk of both depression and anxiety, which confirms previous findings regarding the protective effect of higher levels of education against anxiety and depression (64) as a result of greater upward social mobility (65). A recent study on intergenerational transmission of depression in South Africa (66) reported that due to the historical social exclusion of people of color from access to quality education during the *Apartheid* era (1948–1994), many South African adults are adversely affected by their parents' education. Furthermore, vulnerable populations are at higher risk of adverse mental health due to their poor social environment (67). For example, South African adults living in communities that experience relatively higher levels of social dysfunction (crime) such as those residing in the metropolitan areas, are more likely to experience mental health problems (67, 68) demonstrating that poor mental health, poverty, and socio-economic status remain inextricably linked.

Despite the high prevalence of mental health problems across South Africa, few mental health services exist at primary health care facilities. Our data indicates that one in every four adults are likely to require mental health services, but it has been reported that only one in four South Africans with a severe mental disorder, or 27%, will receive treatment (69). In addition, mental ill-health is unfortunately surrounded by high levels of stigmatism in South Africa (70). This, in combination with the low prioritization of mental health, lends itself to poor implementation of mental health care plans, scarcity of trained mental health professionals and most importantly, a lack of a health care budget targeted at prevention, treatment, and support strategies (71). Although mental health care was considered an essential service during the COVID-19 pandemic lockdown period in South Africa, restrictions on physical contact and in-person consultations, along with transport and financial restrictions made access difficult. Thus, those seeking treatment were unable to access services, resulting in notable decreases in mental health visits (72).

Several countries have effectively integrated mental health services into primary health care facilities, with gains for both the national health care system and their respective patients (73, 74). For instance, Zimbabwe has introduced primary health care services for mental health that utilize health care workers (73), which, for the most part, is beneficial for countries who lack resources, such as South Africa. It is therefore recommended that primary health care facilities provide comprehensive mental health services including prevention, screening, treatment, and support strategies (75), which will likely result in better outcomes for those most susceptible.

A strength of this study was its use of national survey data, including respondents from all nine provinces of South Africa, weighted to be representative of South Africa's adult population. The field staff went through extensive training, but a limitation is that we did not conduct formal between fieldworker validation on all survey items. Self-reported mental health questionnaires where respondents are required to report their own personal experiences pose bias (76). Respondents are more likely to report experiences that are socially acceptable or preferred thus, we acknowledge this as an additional limitation of this study. By including a measure of ACEs, the study provides evidence for the contribution of early adversity to current mental health problems, over and above contemporary socio-demographics.

In conclusion, this study suggests that as many as one quarter of South African adults may suffer from probable depression with higher levels in certain provinces. Our findings also highlight that probable anxiety and ACE exposure differs across the provinces. Both probable depression and probable anxiety were more frequently observed among older, widowed, divorced, or separated adults and those with less education, fewer household assets and/or higher ACE exposure. Increased provision of accessible interventions and counseling programs is recommended especially in provinces with a relatively higher prevalence of mental health problems, with a specific focus on those at greater risk.

## Data availability statement

The original contributions presented in the study are included in the article/Supplementary material, further inquiries can be directed to the corresponding author/s.

## Ethics statement

The studies involving human participants were reviewed and approved by the Human Research Ethics Committee (Non-Medical) of the University of the Witwatersrand, South Africa (H21/06/36). The patients/participants provided their written informed consent to participate in this study.

## Author contributions

SAN was responsible for oversight of data collection. AC carried out the data analyses and generated tables, interpreted the data, did the literature search, and the writing of the paper. All authors were involved in the conception and planning of the study, interpretation of the results, interpreted the data, made a significant contribution to the interpretation of the results, were responsible for revising the manuscript, and approved the submitted version.

## Funding

The financial assistance of the Department of Science and Innovation (DSI) and the National Research Foundation (NRF) toward this research is acknowledged. SAN, LW, and JD were supported by the DSI-NRF Center of Excellence in Human Development at the University of Witwatersrand, Johannesburg.

## Conflict of interest

Author WM was employed by Wits Health Consortium (PTY) Ltd. The remaining authors declare that the research was conducted in the absence of any commercial or financial relationships that could be construed as a potential conflict of interest.

## Publisher's note

All claims expressed in this article are solely those of the authors and do not necessarily represent those of their affiliated organizations, or those of the publisher, the editors and the reviewers. Any product that may be evaluated in this article, or claim that may be made by its manufacturer, is not guaranteed or endorsed by the publisher.

## Author disclaimer

Opinions expressed and conclusions arrived at, are those of the author and are not necessarily to be attributed to the DSI-NRF.

## References

[1] GBD 2019 Disease and Injuries Collaborators. Global burden of 369 diseases and injuries in 204 countries and territories, 1990–2019: a systematic analysis for the Global Burden of Disease Study 2019. Lancet. (2020) 396:1204–22. 10.1016/S0140-6736(20)30925-9 33069326 PMC7567026

[2] The World Bank. Available online at: https://data.worldbank.org/indicator/SI.POV.GINI (accessed July, 4 2022).

[3] Herman AA Stein DJ Seedat S Heeringa SG Moomal H Williams DR. The South African Stress and Health (SASH) study: 12-month and lifetime prevalence of common mental disorders. S Afr Med J. (2009) 99:339–44. 19588796 PMC3191537

[4] Andersson LM Schierenbeck I Strumpher J Krantz G Topper K Backman G. Help-seeking behaviour, barriers to care and experiences of care among persons with depression in Eastern Cape, South Africa. J Affect Disord. (2013) 151:439–48. 10.1016/j.jad.2013.06.022 23890669

[5] Mkhwanazi S Gibbs A. Risk factors for generalized anxiety disorder among young women and men in informal settlements in South Africa: a cross-sectional study. SSM – MH. (2021) 1:100010. 10.1016/j.ssmmh.2021.100010

[6] Jewkes RK Dunkle K Nduna M Jama PN Puren A. Associations between childhood adversity and depression, substance abuse and HIV and HSV2 incident infections in rural South African youth. Child Abuse Negl. (2010) 34:833–41. 10.1016/j.chiabu.2010.05.002 20943270 PMC2981623

[7] Horn PJ Wuyek LA. Anxiety disorders as a risk factor for subsequent depression. Int J Psychiatry Clin Pract. (2010) 14:244–7. 10.3109/13651501.2010.487979 24917433

[8] Barry MM Clarke AM Jenkins R Patel V. A systematic review of the effectiveness of mental health promotion interventions for young people in low and middle-income countries. BMC Public Health. (2013) 13:835. 10.1186/1471-2458-13-835 24025155 PMC3848687

[9] Benjet C. Childhood adversities of populations living in low-income countries: prevalence, characteristics, and mental health consequences. Curr Opin Psychiatry. (2010) 23:356–62. 10.1097/YCO.0b013e32833ad79b 20520546

[10] McGee RE Thompson NJ. Peer-reviewed: unemployment and depression among emerging adults in 12 states, behavioral risk factor surveillance system, 2010. Prev Chronic Dis. (2015) 12:140451. 10.5888/pcd12.140451 25789499 PMC4372159

[11] Lorant V Deliège D Eaton W Robert A Philippot P Ansseau M. Socioeconomic inequalities in depression: a meta-analysis. Am J Epidemiol. (2003) 157:98–112. 10.1093/aje/kwf182 12522017

[12] Akhtar-Danesh N Landeen J. Relation between depression and sociodemographic factors. Int J Ment Health Syst. (2007) 1:4. 10.1186/1752-4458-1-4 18271976 PMC2241832

[13] Ajaero CK Nzeadibe CT Igboeli EE. Rural-urban differences in the prevalence and predictors of depression among adolescents in South Africa. S Afr J Child Health. (2018) 12(Suppl. 2):S71–4. 10.7196/SAJCH.2018.v12i2b.150923236959

[14] Kumar A Nayar KR. COVID 19 and its mental health consequences. J Ment Health. (2021) 30:1–2. 10.1080/09638237.2020.1757052 32339041

[15] Kim AW Nyengerai T Mendenhall E. Evaluating the mental health impacts of the COVID-19 pandemic: perceived risk of COVID-19 infection and childhood trauma predict adult depressive symptoms in urban South Africa. Psychol Med. (2022) 52:1587–99. 10.1017/S0033291720003414 32895082 PMC7520640

[16] Nguse S Wassenaar D. Mental health and COVID-19 in South Africa. S Afr J Psychol. (2021) 51:304–13. 10.1177/00812463211001543 PMC8107260 38603189

[17] Lund C Tomlinson M De Silva M Fekadu A Shidhaye R Jordans M. PRIME: a programme to reduce the treatment gap for mental disorders in five low-and middle-income countries. PLoS Med. (2012) 9:e1001359. 10.1371/journal.pmed.1001359 23300387 PMC3531506

[18] Ridley M Rao G Schilbach F Patel V. Poverty, depression, and anxiety. Casual evidence and mechanisms. Science. (2020) 370:eaay0214. 10.1126/science.aay0214 33303583

[19] Kessler RC Merikangas KR Wang PS. Prevalence, comorbidity, and service utilization for mood disorders in the United States at the beginning of the twenty-first century. Annu Rev Clin Psychol. (2007) 3:137–58. 10.1146/annurev.clinpsy.3.022806.091444 17716051

[20] Gao K Wang Z Chen J Kemp DE Chan PK Conroy CM. Should an assessment of Axis I comorbidity be included in the initial diagnostic assessment of mood disorders? Role of QIDS-16-SR total score in predicting number of Axis I comorbidity. J Affect Disord. (2013) 148:256–64. 10.1016/j.jad.2012.12.004 23273550

[21] Chang X Jiang X Mkandarwire T Shen M. Associations between adverse childhood experiences and health outcomes in adults aged 18-59 years. PLoS ONE. (2019) 14:e0211850. 10.1371/journal.pone.0211850 30730980 PMC6366931

[22] Balen J McManus DP Li YS Zhao ZY Yuan LP Utzinger J. Comparison of two approaches for measuring household wealth *via* an asset-based index in rural and peri-urban settings of Hunan province, China. Emerg Themes Epidemiol. (2010) 7:7. 10.1186/1742-7622-7-7 20813070 PMC2942820

[23] Howe LD Galobardes B Matijasevich A Gordon D Johnston D Onwujekwe O. Measuring socio-economic position for epidemiological studies in low- and middle-income countries: a methods of measurement in epidemiology paper. Int J Epidemiol. (2012) 41:871–86. 10.1093/ije/dys037 22438428 PMC3396323

[24] Montgomery MR Gragnolati M Burke KA Paredes E. Measuring living standards with proxy variables. Demography. (2000) 37:155–74. 10.2307/2648118 10836174

[25] Kroenke K Spitzer RL Williams JBW Löwe B. The patient health questionnaire somatic, anxiety, and depressive symptom scales: a systematic review. Gen Hosp Psychiatry. (2010) 32:345–59. 10.1016/j.genhosppsych.2010.03.006 20633738

[26] Spritzer RL Kroenke K Williams JBW Löwe B. A brief measure for assessing generalized anxiety disorder the GAD-7. Arch Intern Med. (2006) 166:1092–7. 10.1001/archinte.166.10.1092 16717171

[27] Murphy A Steele M Dube SR Bate J Bonuck K Meissner P. Adverse childhood experiences (ACEs) questionnaire and adult attachment interview (AAI): implications for parent child relationships. Child Abus Negl. (2014) 38:224–33. 10.1016/j.chiabu.2013.09.004 24670331

[28] Campbell JA Walker RJ Egede LE. Associations between adverse childhood experiences, high-risk behaviors, and morbidity in adulthood. Am J Prev Med. (2016) 50:344–52. 10.1016/j.amepre.2015.07.022 26474668 PMC4762720

[29] Valladares-Garrido MJ Soriano-Moreno AN Rodrigo-Gallardo PK Moncada-Mapelli E Pacheco-Mendoza J Toro-Huamanchumo CJ. Depression among Peruvian adults with hypertension and diabetes: analysis of a national survey. Diabetes Metab Syndr. (2020) 14:141–6. 10.1016/j.dsx.2020.02.001 32087565

[30] Lopes CS Hellwig N Menezes PR. Inequities in access to depression treatment: results of the Brazilian National Health Survey – PNS. Int J Equity Health. (2016) 15:154. 10.1186/s12939-016-0446-1 27852278 PMC5112732

[31] Onuh JC Mbah PO Ajaero CK Orjiakor CT Igboeli EE Ayogu CK. Rural-urban appraisal of the prevalence and factors of depression status in South Africa. J Affect Disord. (2021) 4:82. 10.1016/j.jadr.2021.100082

[32] Malat J Oh HJ Hamilton MA. Poverty experience, race, and child health. Public Health Rep. (2005) 120:442–7. 10.1177/003335490512000411 16025724 PMC1497743

[33] Whiteside-Mansell L McKelvey L Saccente J Selig JP. Adverse childhood experiences of urban and rural preschool children in poverty. Int J Environ Res Public Health. (2019) 16:2623. 10.3390/ijerph16142623 31340510 PMC6678738

[34] Probst JC Laditka SB Moore CG Harun N Powell MP Baxley EG. Rural-urban differences in depression prevalence: implications for family medicine. Fam Med. (2006) 38:653–60. 17009190

[35] Statistics, South Africa,. National Poverty Line. Available online at: https://www.statssa.gov.za/ (accessed September 29, 2022).

[36] Cummins P. Access to health care in the Western Cape. Lancet. (2002) 360:S49–S50. 10.1016/S0140-6736(02)11820-4 12504503

[37] Maphumulo WT Bhengu BR. Challenges of quality improvement in the healthcare of South Africa post-apartheid: a critical review. Curationis. (2019) 42:e1–9. 10.4102/curationis.v42i1.1901 31170800 PMC6556866

[38] McLaren ZM Ardington C Leibbrandt M. Distance decay and persistent health care disparities in South Africa. BMC Health Serv Res. (2014) 14:541. 10.1186/s12913-014-0541-1 25367330 PMC4236491

[39] Statistics, South Africa,. Stats SA releases Census of Commercial Agriculture 2017 Report. Available online at: https://www.statssa.gov.za (accessed September 29, 2022).

[40] Weiss SJ Simeonova DI Kimmel MC Battle CL Maki PM Flynn HA. Anxiety and physical health problems increase the odds of women having more severe symptoms of depression. Arch Women Ment Hlth. (2016) 19:491–9. 10.1007/s00737-015-0575-3 26403982

[41] Tomlinson M Kleintjes S Lake L. South African Child Gauge 2021/2022. Cape Town: Children's Institute, University of Cape Town (2022).

[42] Rochat T Redinger S. A life-course perspective on the biological, psychological and social development of child mental health. In:Tomlinson M Kleintjes S and Lake L, editors. South African Child Guage 2021/2022. Cape Town: Children's Institute, University of Cape Town (2022).

[43] Chapman DP Whitfield CL Felitti VJ Dube SR Edwards VJ Anda RF. Adverse childhood experiences and the risk of depressive disorders in adulthood. J Affect Disord. (2004) 82:217–25. 10.1016/j.jad.2003.12.013 15488250

[44] Naicker SN Norris SA Richter LM. Secondary analyses of retrospective and prospective reports of adverse childhood experiences and mental health in young adulthood: filtered through recent stressors. E Clin Med. (2021) 40:101094. 10.1016/j.eclinm.2021.101094 34746715 PMC8548929

[45] World Health Organisation. Nurturing Care for Early Childhood Development. Available online at: https://www.who.int/teams/maternal-newborn-child-adolescent-health-and-ageing/child-health/nurturing-care (accessed September 30, 2022).

[46] Anthony JC Petronis KR. Suspected risk factors for depression among adults 18–44 years old. Epidemiology. (1991) 2:123–32. 10.1097/00001648-199103000-00006 1932309

[47] Wade TJ Cairney J. The effect of sociodemographics, social stressors, health status and psychosocial resources on the age-depression relationship. Can J Pub Health. (2000) 91:307–12. 10.1007/BF03404295 10986792 PMC6980130

[48] Aseltine RH Kessler RC. Marital disruption and depression in a community sample. J Health Soc Behav. (1993) 34:237–51. 10.2307/2137205 7989668

[49] Menaghan EG Lieberman MA. Changes in depression following divorce: a panel study. J Marriage Fam. (1986) 48:319–28. 10.2307/352399

[50] Richards M Hardy R Wadsworth M. The effects of divorce and separation on mental health in a national UK birth cohort. Psychol Med. (1997) 27:1121–8. 10.1017/S003329179700559X 9300516

[51] Roterman M. Marital breakdown and subsequent depression. Health Rep. (2007) 18:33–44. 17578014

[52] Bulloch AG Williams JV Lavorato DH Patten SB. Relationship between major depression and marital distribution is bidirectional. Depress Anxiety. (2009) 26:1172–7. 10.1002/da.20618 19798680

[53] Blazer DG. Depression in late life: review and commentary. J Gerontol A Biol Sci Med Sci. (2003) 58:249–65. 10.1093/gerona/58.3.M249 12634292

[54] Wang JK Su TP Chou P. Sex differences in prevalence and risk indicators of geriatric depression: the Shih-Pai community-based survey. J Formos Med Assoc. (2010) 109:345–53. 10.1016/S0929-6646(10)60062-9 20497867

[55] Yu J Li J Cuijpers P Wu S Wu Z. Prevalence and correlates of depressive symptoms in Chinese older adults: a population-based study. Int J Geriatr Psychiatr. (2012) 27:305–12. 10.1002/gps.2721 21538538

[56] Blazer D. Psychiatry and the oldest old. Am J Psychiatr. (2000) 157:1915–24. 10.1176/appi.ajp.157.12.1915 11097951

[57] Blazer D Moody-Ayers S Craft-Morgan J Burchett B. Depression in diabetes and obesity: racial/ethnic/gender issues in older adults. J Psychosom Res. (2002) 52:1–4. 10.1016/S0022-3999(02)00314-8 12377303

[58] Robinson R Price T. Post-stroke depressive disorders: a follow-up study of 103 patients. Stroke. (1982) 13:635–41. 10.1161/01.STR.13.5.635 7123596

[59] Weiner M Edland S Luszczynska H. Prevalence and incidence of major depression in Alzheimer's Disease. Am J Psychiatr. (1994) 151:1006–9. 10.1176/ajp.151.7.1006 8010355

[60] Zunzunegui MV Béland F. Gac Sanit. *I*ntersectoral policies to meet the challenge of active aging. SESPAS Rep. (2010) 24:68–73. 10.1016/j.gaceta.2010.08.004 21051121

[61] Pabón-Carrasco M Ramirez-Baena L López Sánchez R Rodríguez-Gallego I Suleiman-Martos N Gómez-Urquiza JL. Prevalence of depression in retirees: a meta-analysis. Healthcare. (2020) 8:321. 10.3390/healthcare8030321 32899813 PMC7551681

[62] Aguilera MÁ. Health and Retirement. Guadalajara: University Editorial. (2010).

[63] Nivardo F Gainza V. Retirement ratings. Import Adv Prep Geroinfo. (2009) 4:1–12.

[64] Bjelland I Krokstad S Mykletun A Dahl AA Tell SG Tambs K. Does a higher education level protect against anxiety and depression? The HUNT study. Soc Sci Med. (2008) 66:1334–45. 10.1016/j.socscimed.2007.12.019 18234406

[65] Mungai K Bayat A. An overview of trends in depressive symptoms in South Africa. S Afr J Psychol. (2019) 49:1–18. 10.1177/008124631882358034240393

[66] Eyal K Burns J. The Intergenerational Transmission of Depression in South African Adolescents (Southern Africa Labour and Development Research Unit, Working paper no. 200). Cape Town: SALDRU, University of Cape Town.

[67] Tomita A Labys CA Burns JK. A multilevel analysis of the relationship between neighborhood social disorder and depressive symptoms: evidence from the South African National Income Dynamics Study. A J Orthopsychiatr. (2015) 85:56–62. 10.1037/ort0000049 25642654 PMC4322132

[68] Stafford M Chandola T Marmot M. Association between fear of crime and mental health and physical functioning. Am J Public Health. (2007) 97:2076–81. 10.2105/AJPH.2006.097154 17901443 PMC2040373

[69] Pillay Y. State of mental health and illness in South Africa. S Afr J Psychiatr. (2019) 49:463–6. 10.1177/0081246319857527

[70] Corrigan P. How stigma interferes with mental health care. Am Psychologist. (2004) 59:614–25. 10.1037/0003-066X.59.7.614 15491256

[71] Marais DL Petersen I. Health system governance to support integrated mental health care in South Africa: challenges and opportunities. IJMHS. (2015) 9:1–21. 10.1186/s13033-015-0004-z 25806085 PMC4372271

[72] Pillay AL Barnes BR. Psychology and COVID-19: impacts, themes and way forward. S Afr J Psychol. (2020) 50:148–53. 10.1177/0081246320937684

[73] Chibanda D Weiss HA Verhey R Simms V Munjoma R Rusakaniko S. Effect of a primary care–based psychological intervention on symptoms of common mental disorders in Zimbabwe: a randomized clinical trial. JAMA. (2016) 316:2618–26. 10.1001/jama.2016.19102 28027368

[74] Buttorff C Hock RS Weiss HA Naik S Araya R Kirkwood BR Chisholm D Patel V. Economic evaluation of a task-shifting intervention for common mental disorders in India. Bull World Health Org. (2012) 90:813–21. 10.2471/BLT.12.104133 23226893 PMC3506405

[75] Francis E Bowers KS Buchberger G Ryan S Milchak W Kraschnewski J. Reducing alcohol and opioid use among youth in rural counties: an innovative training protocol for primary health care providers and school personnel. JMIR Res Protoc. (2020) 9:e21015. 10.2196/21015 33155572 PMC7679207

[76] Devaux M Sassi F. Social disparities in hazardous alcohol use: self-report bias may lead to incorrect estimates. Eur J Public Health. (2016) 26:129–34. 10.1093/eurpub/ckv190 26585784 PMC4851717

