# Supplementary material for: The prevalence of probable depression and probable anxiety, and associations with adverse childhood experiences and socio-demographics: A national survey in South Africa
Source: Front Public Health. 2022 Oct 28;10:986531. doi: 10.3389/fpubh.2022.986531 (PMC9650309; doi:10.3389/fpubh.2022.986531)
Supplement: Supplementary file 1 [file Data_Sheet_1.docx]

**Supplementary Table S1. Adverse childhood event questionnaire and categories**

| Category | Question |
| --- | --- |
| **Emotional/ physical abuse** | 1. Did a parent or other adult in the household often or very often swear at you, insult you, put you down, or humiliate you? 2. Did you often or very often feel that no one in your family loved you or thought you were important or special? 3. Did you often or very often feel that you didn’t have enough to eat, had to wear dirty clothes, and had no one to protect you? 4. Did either of your parents/caregivers pass away before you turned 18? 5. Did a parent or other adult in the household often or very often push, grab, slap, or throw something at you? |
| **Household dysfunction** | 1. Was your mother or stepmother often or very often pushed, grabbed, slapped, or had something thrown at her? 2. Did you live with anyone who was a problem drinker or alcoholic or used street drugs? 3. Was a household member depressed or mentally ill, or did a household member attempt suicide? 4. Did a household member go to prison? 5. Was there anyone in your household who was chronically ill when you were a child? 6. Was one or more of your parents/caregivers mostly unemployed during your childhood because they could not get a job? |
| **Sexual abuse** | 1. Did an adult or person at least 5 years older than you ever touch or fondle you or have you touch their body in a sexual way? |

**Supplementary Table S2. Mental health risk of the South African adult population stratified by province.**

|  | | | **Provinces** | | | | | | | | |
| --- | --- | --- | --- | --- | --- | --- | --- | --- | --- | --- | --- |
|  | | | **WC** | **EC** | **NC** | **FS** | **KZN** | **NW** | **GP** | **MP** | **LP** |
| **Depression (PHQ9)** | **Probable depression** | % | **31.8** | **34.7** | **38.8** | **14.7** | **14.9** | **22.1** | **29.2** | **25.1** | **21.3** |
|  | Minimal depression | % | 40.2 | 36.2 | 57.6 | 55.0 | 56.7 | 43.4 | 49.0 | 51.9 | 50.1 |
|  | Mild depression | % | 28.0 | 29.1 | 3.6 | 30.3 | 28.4 | 34.5 | 21.8 | 23.0 | 28.7 |
|  | Moderate depression | % | 19.2 | 26.7 | 7.8 | 9.0 | 11.7 | 17.5 | 17.0 | 15.5 | 17.1 |
|  | Moderately severe depression | % | 9.6 | 8.0 | 19.9 | 4.4 | 2.7 | 4.6 | 9.8 | 5.7 | 2.3 |
|  | Severe depression | % | 3.0 | 0.0 | 11.1 | 1.3 | 0.5 | 0.0 | 2.4 | 3.9 | 1.8 |
| **Anxiety (GAD7)** | **Probable anxiety** | **%** | **23.9** | **22.0** | **29.3** | **13.0** | **8.6** | **10.4** | **22.2** | **14.6** | **12.1** |
|  | Minimal anxiety | % | 45.9 | 39.7 | 59.4 | 59.9 | 70.8 | 63.8 | 56.8 | 66.4 | 59.4 |
|  | Mild anxiety | % | 30.2 | 38.3 | 11.3 | 27.1 | 20.6 | 25.8 | 21.0 | 19.0 | 28.5 |
|  | Moderate anxiety | % | 16.7 | 20.7 | 13.2 | 12.4 | 6.6 | 10.0 | 17.2 | 10.8 | 11.6 |
|  | Severe anxiety | % | 7.2 | 1.3 | 16.1 | 0.6 | 2.0 | 0.4 | 5.0 | 3.8 | 0.5 |
| **ACEs** | ACE score | Mean ± SD | 2.7 ± 2.9 | 2.7 ± 27 | 1.7 ± 2.4 | 1.9 ± 3.3 | 1.6 ± 2.5 | 1.5 ± 2.3 | 1.9 ± 2.5 | 2.3 ± 2.4 | 1.5 ± 2.5 |

Abbreviations: %: percentage; ACEs: adverse childhood experiences; PHQ9: Patient Health Questionnaire; GAD7: Generalised Anxiety Disorder; WC: Western Province; EC: Eastern Cape; NC: Northern Cape; FS: Free State; KZN: Kwa-Zulu Natal; NW: North West Province; GP: Gauteng Province; MP: Mpumalanga; LP: Limpopo Province.

**Supplementary Table S3. Socio demographics of the South African adult population stratified by mental health risk.**

|  |  | **Generalised Anxiety Disorder (GAD7)** | | | | | **Patient Health Questionnaire (PHQ9)** | | | | | | **ACEs** | | |
| --- | --- | --- | --- | --- | --- | --- | --- | --- | --- | --- | --- | --- | --- | --- | --- |
|  |  | **Probable anxiety** | **Min** | **Mild** | **Moderate** | **Severe** | **Probable depression** | **Min** | **Mild** | **Moderate** | **Moderately severe** | **Severe** | **Low exposure** | **Intermediate exposure** | **High exposure** |
| **Age** | | | | | | | | | | | | | | | |
| 18-24 years | % | **15.8** | 57.1 | 27.0 | 11.6 | 4.2 | **24.8** | 45.7 | 29.5 | 15.3 | 6.7 | 2.8 | 46.8 | 33.5 | **19.7** |
| 25-34 years | % | **15.8** | 61.5 | 22.7 | 12.0 | 3.8 | **22.8** | 50.7 | 26.5 | 15.5 | 5.6 | 1.7 | 47.2 | 30.5 | **22.3** |
| 35-44 years | % | **17.6** | 52.4 | 30.0 | 14.8 | 2.8 | **26.9** | 45.7 | 27.4 | 18.2 | 6.8 | 1.9 | 48.9 | 26.8 | **24.2** |
| 45-54 years | % | **20.9** | 58.4 | 20.7 | 16.1 | 4.8 | **26.6** | 49.6 | 23.8 | 18.0 | 7.6 | 1.0 | 42.4 | 29.4 | **28.2** |
| 55-64 years | % | **18.5** | 63.4 | 18.2 | 15.9 | 2.6 | **24.5** | 52.2 | 23.2 | 11.6 | 12.2 | 0.7 | 50.9 | 27.9 | **21.2** |
| 65+ years | % | **22.6** | 49.0 | 28.5 | 19.7 | 2.9 | **39.0** | 43.5 | 17.5 | 29.9 | 3.9 | 5.2 | 43.7 | 32.6 | **23.7** |
| **Sex** | | | | | | | | | | | | | | | |
| Male | % | **17.1** | 58.9 | 24.0 | 13.5 | 3.6 | **24.6** | 50.2 | 25.1 | 16.0 | 7.2 | 1.4 | 48.0 | 28.5 | **23.6** |
| Female | % | **18.3** | 55.7 | 25.9 | 14.8 | 3.5 | **26.7** | 46.2 | 27.1 | 17.7 | 6.8 | 2.2 | 46.0 | 30.3 | **23.7** |
| **Marital status** | | | | | | | | | | | | | | | |
| Single | % | **17.4** | 55.4 | 27.2 | 14.1 | 3.3 | **25.6** | 45.9 | 28.6 | 16.9 | 6.9 | 1.8 | 45.9 | 30.4 | **23.7** |
| Married/Co-habit | % | **17.5** | 61.0 | 21.5 | 13.5 | 4.0 | **23.8** | 52.5 | 23.6 | 15.5 | 7.1 | 1.2 | 48.0 | 26.9 | **25.1** |
| Widowed/Divorced | % | **20.4** | 56.2 | 23.3 | 16.8 | 3.6 | **32.6** | 47.1 | 20.2 | 21.4 | 7.5 | 3.7 | 49.7 | 32.2 | **18.0** |
| **Employment** | | | | | | | | | | | | | | | |
| Unemployed | % | **21.9** | 51.6 | 26.6 | 17.6 | 4.3 | **29.9** | 41.3 | 28.7 | 18.2 | 9.1 | 2.6 | 41.1 | 28.2 | **30.8** |
| Employed | % | **16.4** | 59.2 | 24.5 | 13.0 | 3.4 | **23.3** | 51.2 | 25.4 | 16.1 | 6.1 | 1.1 | 48.5 | 29.6 | **21.9** |
| Student | % | **14.6** | 63.2 | 22.3 | 10.9 | 3.7 | **25.4** | 47.9 | 26.7 | 17.3 | 5.6 | 2.5 | 51.5 | 34.7 | **13.8** |
| Retired | % | **16.3** | 59.0 | 24.7 | 14.3 | 2.0 | **30.6** | 49.5 | 19.9 | 18.3 | 7.9 | 4.4 | 54.3 | 27.1 | **18.7** |
| **Education** | | | | | | | | | | | | | | | |
| Uneducated/Partial primary | % | **18.0** | 42.7 | 39.3 | 17.4 | 0.6 | **28.3** | 33.6 | 38.1 | 22.5 | 3.3 | 2.5 | 37.3 | 29.8 | **32.9** |
| Primary school | % | **20.4** | 61.3 | 18.3 | 16.8 | 3.6 | **32.1** | 39.4 | 28.6 | 12.3 | 10.8 | 9.0 | 29.3 | 35.6 | **35.1** |
| Partial secondary | % | **18.0** | 52.3 | 29.7 | 14.5 | 3.5 | **26.5** | 44.7 | 28.7 | 17.6 | 7.2 | 1.7 | 33.0 | 34.3 | **32.7** |
| NSC/Short course | % | **18.1** | 58.0 | 23.9 | 14.1 | 4.0 | **27.3** | 47.4 | 25.3 | 17.8 | 7.4 | 2.1 | 47.0 | 30.1 | **22.9** |
| Tertiary | % | **16.8** | 60.1 | 23.1 | 13.7 | 3.1 | **21.9** | 53.2 | 24.8 | 14.8 | 6.2 | 0.9 | 57.6 | 24.7 | **17.7** |
| **Urbanicity** | | | | | | | | | | | | | | | |
| Metropolitan | % | **19.7** | 56.0 | 24.2 | 15.4 | 4.3 | **27.0** | 49.4 | 23.6 | 17.4 | 8.0 | 1.6 | 49.4 | 27.3 | **23.3** |
| City/Town | % | **15.6** | 60.4 | 24.0 | 11.5 | 4.1 | **22.4** | 50.6 | 26.9 | 13.8 | 5.1 | 3.5 | 46.2 | 30.6 | **23.2** |
| Rural/Village | % | **15.1** | 57.2 | 27.6 | 13.9 | 1.2 | **25.9** | 42.8 | 31.3 | 18.7 | 6.6 | 0.6 | 41.9 | 33.2 | **24.9** |
| **Household assets** | | | | | | | | | | | | | | | |
| Lower tertile | % | **17.7** | 55.2 | 27.0 | 15.1 | 2.6 | **26.4** | 43.5 | 30.1 | 18.7 | 6.3 | 1.4 | 40.3 | 30.8 | **28.9** |
| Middle tertile | % | **18.0** | 53.8 | 28.2 | 14.6 | 3.4 | **26.3** | 47.0 | 26.6 | 17.0 | 7.9 | 1.4 | 45.2 | 32.1 | **22.7** |
| Upper tertile | % | **17.4** | 64.0 | 18.5 | 12.5 | 4.9 | **24.2** | 55.2 | 20.7 | 14.7 | 6.8 | 2.7 | 56.9 | 24.6 | **18.5** |

Abbreviations: %: percentage; ACEs: adverse childhood experiences; PHQ9: Patient Health Questionnaire; GAD7: Generalised Anxiety Disorder.
